# A 15q25.2 microdeletion phenotype for premature ovarian failure in a Chinese girl: a case report and review of literature

**DOI:** 10.1186/s12920-020-00787-w

**Published:** 2020-09-07

**Authors:** Zhen Chen, Hong Chen, Ke Yuan, Chunlin Wang

**Affiliations:** 1 Department of Pathology, The First Affiliated Hospital of Medical College, Zhejiang University, Hangzhou, 310003 Zhejiang province China; 2 Department of Pediatrics, The First Affiliated Hospital of Medical College, Zhejiang University, Hangzhou, 310003 Zhejiang province China

**Keywords:** 15q25.2, Microdeletion, Premature ovarian failure, BCN1 gene

## Abstract

**Background:**

Proximal microdeletions on chromosome 15q25.2 are very rare, and are associated with neurodevelopmental delay, inguinal hernia, chest deformities, and anemia. The minimum length missed so far is 1.4 Mb. However, there were no cases reported till date on microdeletions at position q25.2 on chromosome 15 with premature ovarian failure (POF).

**Case presentation:**

We herein reported a POF case characterized by short stature with only 0.447 Mb deletion on chromosome 15q25.2. The clinical and molecular characteristics in our patient showed the slightest clinical manifestations, with no clinical signs of neurodevelopmental delay, inguinal hernia, chest deformities, and anemia when compared to the previously reported cases. The microdeletions in our case included only 7 genes (HOMER2, FAM103A1, C15orf40, BTBD1, TM6SF1, HDGFRP3 and BNC1), and excluded the CPEB1 gene. Among these, the BNC1 gene is the only one that is known to be involved in reproduction. We hypothesized that the deletion of BNC1 gene in this patient led to haploinsufficiency, and consequently to POF.

**Conclusions:**

The study of this case increased the knowledge on the molecular and phenotypic consequences of interstitial 15q25.2 deletion, emphasizing that BNC1 gene deletion in this region might contribute to POF.

## Background

Premature ovarian failure (POF) is a complex heterogeneous disease and a common genetic condition affecting 1 ~ 1.5% of women under 40 years of age, and the main manifestations include abnormal menstruation (amenorrhea, oligomenorrhea or frequent), elevated levels of gonadotropin (FSH > 25 U/L), and decreased female hormone level volatility. But in 50–80% of cases, POF is still classified as being idiopathic, suggesting a strong genetic association of the disease [1]. In some cases, POF is caused by single-gene mutations that are inherited by autosomes or X-linked, while it is caused by chromosomal abnormalities, such as Turner syndrome, in few others [2]. DNA microarray technology is used for detecting the submicroscopic copy number variation (CNV) revealing the pathogenesis of rare and complex diseases. The chromosome 15q25.2 region has a complex genomic structure and four low-copy repeats, which mediate non-allelic homologous recombination and result in the deletion of chromosome fragments [3]. Till date, there are about 10 patients with proximal 15q25.2 microdeletions reported [3–6]. The clinical phenotypic features of these patients include retarded intellectual development, short stature, craniofacial deformities, congenital diaphragmatic hernia (CDH), Diamond-Blackfan anemia (DBA), and mental disorders. In addition, some rare phenotypes included cleft lip, dextrocardia, and obstructive sleep apnea [3–8]. The clinical phenotype of these patients showed close association with the genes present in the deletion region. However, there are few reports on 15q25.2 microdeletions and POF.

We herein reported a case with 15q25.2 microdeletions and compared this patient with the previously reported female cases to propose the possible candidate genes for POF based on the observed characteristics.

## Case presentation

A 14-year-9-month-old female who is the only child of healthy and nonconsanguineous Chinese parents was referred to our hospital for investigation due to her short stature and lack of menstruation. At birth, her weight was 3100 g and length was 50 cm. She was born without any difficulty during delivery and her mental and motor abilities remained normal. She developed breasts when she was 13 years old but not menstruated yet. Physical examination revealed a height and weight of 143.9 cm (− 2.9SD) and 29.5 Kg (− 2.98SD), respectively. Karyotype analysis showed 46, XX. The breast development was stage B3 (with obvious enlargement and elevation of the whole breast) and pubic hair development was stage PH4 (the publc hair was long and dark and spread to the pubic region). The bone age is about 12 years old (Gruelich-Pyle). Pelvic ultrasound showed that the uterus size was about 2.5 cm × 2.1 cm × 2.7 cm; and the ovary was not clearly seen. There were no abnormalities detected in the ultrasound results of urinary and cardiovascular systems. The baseline levels of reproductive hormones revealed LH of 10.40 IU/L (normal: 0.00–15.26), FSH of 45.5 IU/L (normal: 0.19–7.97), and E2 of 18.3 pmol/L (normal: 26.9–254.6).

**Fig. 1:**
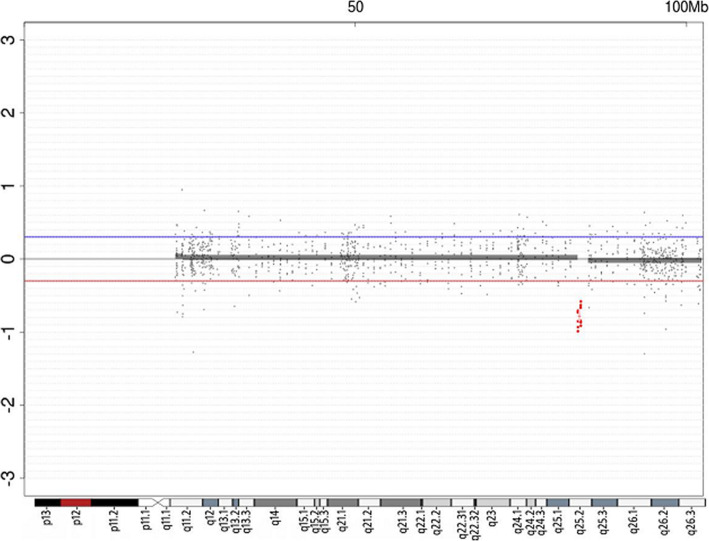
DNA microarray profiles of the patient. The horizontal axis is the chromosome number, and the vertical axis is the signal ratio between the sample and the standard sample. The profile of Agilent array CGH showed a Log ratio of −1, which indicated a heterozygous deletion

**Fig. 2:**
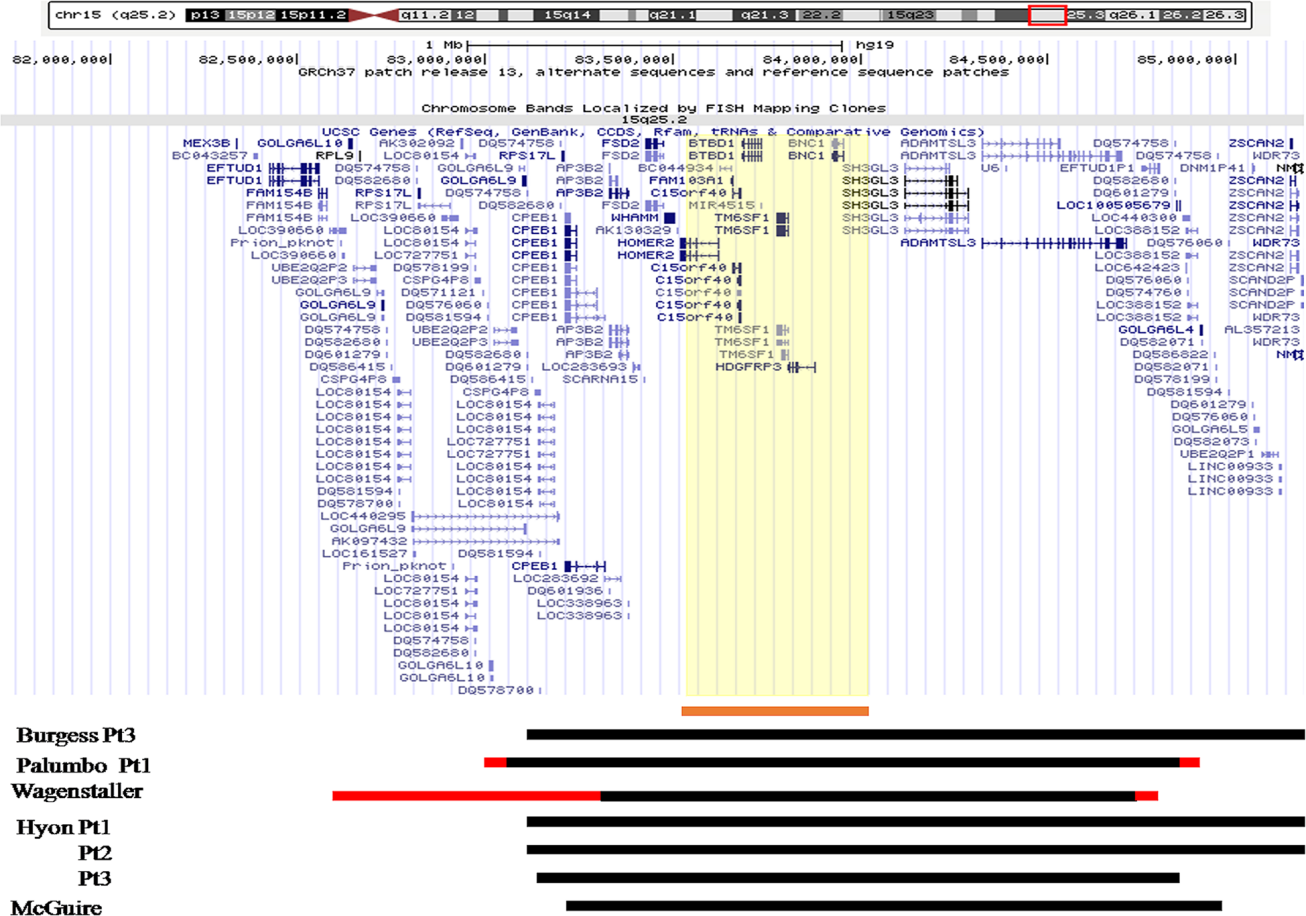
Schematic representation of chromosome region 15q25.2 using UCSC Genome Browser assembly February 2009 hg19. The missing areas in our patient were 15q25.2 83,588,055-84,035,418 when compared to female patients who were reported to have lost the same area. The minimum deleted area in each patient is indicated by a solid black bar and the largest deleted area is indicated in red color. The yellow area represents the missing area in this patient

Based on the breast development, short stature, amenorrhea and undetected ovaries in the 14-year-9-month girl, the baseline levels of LH and FSH were significantly increased, diagnosing POF. DNA microarray analysis (CytoOneArray chip from Phalanx Biotech company) showed a 15q25.2 heterozygous microdeletion from 83,588,055 to 8,403,418 (Fig. 1). The deletion of 15q25.2 includes 7 genes: HOMER2, FAM103A1, C15orf40, BTBD1, TM6SF1, HDGFRP3 and BNC1. The aberration was neither detected in her father nor her mother, indicating that the deletion was arisen de novo. Genomic microdeletion involving chromosome 15q25.2 is a rare finding. Nevertheless, there are some reports of CNVs within this region that are associated with recognizable phenotypes. A search was performed in the NCBI and DECIPHER database (https://decipher.sanger.ac.uk/) and identified 7 patients carrying similar microdeletions. Figure 2 showed the size and location of the 15q25.2 deletion (chr15: 83588055–84,035,418) as compared to the published female cases.

Estrogen replacement therapy is generally used for patients with POF. However, the patient did not receive further treatment because of her parents’ concern regarding the side effects.

## Discussion and conclusions

The chromosome 15q25.2 region has a complex genomic structure and four low-copy repeats that mediate non-allelic homologous recombination and result in deletion of chromosome fragments [3]. The clinical phenotype of these patients is closely related to the genes present in the deleted regions. The clinical phenotypes mainly included mental retardation, short stature, craniofacial deformity, CDH, DBA, and mental disorders. In addition, all female patients have POF [9]. Our patient presented with characteristics of short stature and ovarian dysplasia, without intellectual disability, anemia, structural malformation of the thorax and upper abdomen, venous thrombosis or other clinical manifestations. As reported previously, CPEB1 (cytoplasmic polyadenylation element-binding protein-1, OMIM 607342) is highly expressed in the brain and plays an important role in maintaining the production of mitochondrial ATP in nerve cells. Adaptor protein-3 (AP3) is a heterotetrameric vesicle-coat protein complex that constitutes AP3 complex subunit beta-2 (AP3B2, OMIM 602166) as a subunit of non-clathrin and clathrin-associated AP3, which plays a role in protein sorting in the late-Golgi/trans-Golgi network (TGN) and/or endosomes. AP3 is involved in the sorting of a subset of transmembrane proteins targeted to lysosomes and lysosome-related organelles. Therefore, AP3B2 is thought to have neuro-specific functions, such as releasing of the neurotransmitters. AP3 is a heterotetrameric vesicle-coat protein complex, and so deletion of *CPEB1* gene and *AP3B2* gene is related to the phenotype of neural abnormality, and our patient’s microdeletion does not include these two genes, explaining as to why she has no clinical manifestations of the nervous system. It is currently believed that deletion of *RPS17L* (OMIM 180472) gene might be responsible for the cause of anemia in patients with a proximal 15q25.2 microdeletion, and no loss of this gene has occurred in this patient. BTBD1 (protein 1 containing BTB/POZ domain, OMIM 608530), HDGFRP3 (hepatoma derived growth factor-associated protein 3, OMIM 616643) and BCN1 (basculin 1, OMIM 601930) played roles in cell proliferation [10]. Wat et al. [5] have suggested that these genes were associated with short stature and developmental delay in patients with 15q25.2 deletion.

Hyon et al. [9] have reported three patients with 15q25.2 microdeletions that are associated with ovarian insufficiency. Their minimum common area (chr15:83223426 to 84,832,932) included 12 genes (*CPEB1, AP3B2, FSD2, WHAMM, HOMER2, FAM103A1, C15orf40, BTBD1, TM6SF1, HDGFRP3, BNC1, SH3GL3*), and the heterozygous deletion of *CPEB1* genes was believed to be responsible for the early POF in females [9]. The clinical features of female patients with 15q25.2 deletion are summarized in Table 1. However, the microdeletions in our patient included only 7 genes (*HOMER2, FAM103A1, C15orf40, BTBD1, TM6SF1, HDGFRP3* and *BNC1*), which excluded the *CPEB1* gene (chr15:83211951–83,317,612). Among these, the *BNC1* gene is the only one known to be involved in reproduction. Human *BNC1* gene encodes zinc finger protein basonuclin-1 [11]. *BNC1* is a zinc finger protein that is present in the epidermal basal cell layer and hair follicles. It is also abundantly found in the germ cells of the testes and ovaries. Furthermore, it plays a regulatory role in keratinocyte proliferation, and might also act as a regulator of rRNA transcription. Wittgenstein et al. believed that BNC1 gene is involved in oogenesis and is closely related to POF [12]. *BNC1* as a transcription factor is involved in oogenesis and folliculogenesis [13]. Knockout of *BNC1* gene resulted in female infertility [14]. Recent study found that knockdown of *BNC1* gene can reduce BMP15 and p-akt levels and inhibit meiosis in the oocytes. Female mouse models with Bnc1 mutation showed similar phenotype as that of POF [15]. Zhang et al. have reported a familial POI caused by *BNC1* mutation [15]. The patients with POF reported by Hyon et al. missed both *CPEB1* and *BNC1* genes, and believed that *CPEB1* is the cause of POF. However, the importance of *BNC1* gene in the occurrence and development of POF was proved in our case. Therefore, we hypothesized that deletion of *BNC1* gene in this patient led to haploinsufficiency and consequently to POF.

**Table 1:** Clinical features and molecular breakpoints of patients with 15q25.2 microdeletions

	Burgess Pt3 [6–8]	Palumbo Pt1 [16]	Wagenstaller [4]	Hyon Pt1 [9]	Hyon Pt2 [9]	Hyon Pt3 [9]	McGuire Pt87 [10]	Present case
Age (years)	5-month old	9	11	NR	NR	NR	NR	14
Gender	Female	Female	Female	Female	Female	Female	Female	Female
Height (cm) /weight (kg)	NR	120 /22	NR	168/70	156/48	156/59	NR	143.9/29.5
First menses	NA	NR	NR	Primary amenorrhea	19	Primary amenorrhea	Primary amenorrhea	Primary amenorrhea
Size of the ovary (right/left), mm	NA	NR	NR	Not found	19 × 12/ 20× 11	25 × 8/ 26 × 9	NR	Not found
FSH (IU/L)/ LH (IU/L) / E2(pmol/L)	NR	NR	NR	33/29/39	44/19/90	65/48/100	NR	10.4/45.5/18.3
Intellectual disability or developmental delay	Mild delay	moderate to severe cognitive delay	Mild mental and psychomotor retardation	Behavioral disorders, progressive intellectual deficiency	None	None	NR	None
Microdeletion in 15q25.2	2.5 Mb	1.6 Mb	~ 1.4 Mb/~ 2.2 Mb	2.5 Mb	2.5 Mb	1.6 Mb	1.67 Mb	0.447 Mb
Inheritance	De novo	De novo	De novo	De novo	NR	NR	NR	De novo

*FSH* Follicle stimulating hormone, *LH* Luteinizing hormone, *E2* Estradiol, *NR* Not recorded, *NA* Not applicable, *UNK* Unknown, *N* Normal

In summary, this study extended a relatively minor phenotype of the proximal deletion of chromosome 15q25.2 with POF, and revealed that *BNC1* gene might be an important candidate gene for POF.

## Data Availability

The datasets analyzed during the current study are available from the corresponding author on reasonable request.

## Abbreviations

POF: Premature ovarian failure
CMA: Chromosomal microarray chip
AP3: Adaptor protein-3
CNV: Copy number variation
BNC1: Basonuclin 1
CDH: Congenital diaphragmatic hernia
